# Estimating the spatial risk of tuberculosis distribution in Gurage zone, southern Ethiopia: a geostatistical kriging approach

**DOI:** 10.1186/s12889-018-5711-3

**Published:** 2018-06-25

**Authors:** Sebsibe Tadesse, Fikre Enqueselassie, Seifu Hagos Gebreyesus

**Affiliations:** 1 Institute of Public Health, College of Medicine and Health Sciences, University of Gondar, Gondar, Ethiopia; 2 School of Public Health, College of Health Sciences, Addis Ababa University, Addis Ababa, Ethiopia

**Keywords:** Geostatistical kriging, Risk of tuberculosis, Semivariogram model, Spatial heterogeneity

## Abstract

**Background:**

In low-income countries it is difficult to obtain complete data that show spatial heterogeneity in the risk of tuberculosis within-and-between smaller administrative units. This may contribute to the partial effectiveness of tuberculosis control programs. The aim of this study was to estimate the spatial risk of tuberculosis distribution in Gurage Zone, Southern Ethiopia using limited spatial datasets.

**Methods:**

A total of 1601 patient data that were retrieved from unit tuberculosis registers were included in the final analyses. The population and geo-location data were obtained from the Central Statistical Agency of Ethiopia. Altitude data were extracted from ASTER Global Digital Elevation Model Version 2. Aggregated datasets from sample of 169(40%), 254(60%) and 338(80%) kebeles were used to estimate the spatial risk of TB distribution in the Gurage Zone by using a geostatistical kriging approach. The best set of input parameters were decided based on the lowest prediction error criteria of the cross-validation technique. ArcGIS 10.2 was used for the spatial data analyses.

**Results:**

The best semivariogram models were the Pentaspherical, Rational Quadratic, and K-Bessel for the 40, 60 and 80% spatial datasets, respectively. The predictive accuracies of the models have improved with the true anisotropy, altitude and latitude covariates, the change in detrending pattern from local to global, and the increase in size of spatial dataset. The risk of tuberculosis was estimated to be higher at western, northwest, southwest and southeast parts of the study area, and crossed between high and low at west-central parts**.**

**Conclusion:**

This study has underlined that the geostatistical kriging approach can be applied to estimate the spatial risk of tuberculosis distribution in data limited settings. The estimation results may help local public health authorities measure burden of the disease at all locations, identify geographical areas that require more attention, and evaluate the impacts of intervention programs.

**Electronic supplementary material:**

The online version of this article (10.1186/s12889-018-5711-3) contains supplementary material, which is available to authorized users.

## Background

Tuberculosis (TB) continues to place an extraordinary public health, financial and social burden on those afflicted by the disease and their families, and on government. In 2016, there were an estimated 10.4 million incident cases and 1.7 million deaths worldwide [1]. The global distribution of the disease is skewed heavily toward low-and-middle income countries, which accounted for about 87% of all estimated incident cases. Ethiopia is a low-income country in east Africa that remains highly afflicted by TB and is ranked among the list of 14 countries with high burden of TB, Human Immunodeficiency Virus (HIV)-associated TB (TB/HIV) and Drug Resistant TB (DR-TB) [1].

It is difficult to obtain spatially complete data on TB in Ethiopia [1, 2]. About 36% of the estimated TB cases were not notified to the national TB program in 2016. The national TB prevalence surveys were conducted through sampling a limited number of locations due to logistical and financial limitations [3, 4]. Moreover, other regional reports did also not show continuous spatial distribution and burden of the disease within-and-between smaller geographical locations [5, 6]. This presents a substantial obstacle to measure burden of the disease, identify high-risk geographical locations, evaluate the impacts of intervention programs and allocate public health resources.

Spatial interpolation is the process of estimating values for a variable of interest at unmeasured locations using data from the surrounding locations [7]. All the spatial interpolation models share a common underlying assumption of the closer values are more related than the distant ones [8]. These models are divided into two categories, deterministic and geostatistical models [9]. The deterministic models estimate between measured values using mathematical formulas that vary the smoothness of the estimated surface. The spatial correlation of the data is not considered in the estimation. Consequently, deterministic models do not estimate the uncertainty of predictions. Conversely, geostatistical models consider the spatial correlation of the dataset. Rather than giving interpolation weights based on arbitrary formulas, the semivariogram models are used to found out weights from the observed data [10]. The weights dictate how each measured value contributes to the interpolated value at unmeasured location. Geostatistical models produce prediction estimates and associated prediction errors at all unmeasured locations. Kriging is the most robust and widely used geostatistical method of interpolation in many fields of science [11]. It is known as the optimal interpolation method because it minimizes the mean square error of predictions and is statistically unbiased (i.e., estimated values and measured values agree on average) [12]. Recently, there have been several applications of kriging in the area of public health for estimating the predicted risk surface of infectious diseases, such as TB [3], malaria [11], cholera [13], helminths [14] and schistosomiasis [15].

The aim of this study was to estimate the spatial risk of TB distribution in Gurage Zone, Southern Ethiopia. Three geostatistical kriging models were fitted with 40, 60 and 80% of spatially aggregated TB dataset co-impacted by geographical factors. The estimated risk map may help local authorities as a guide for planning, budgeting and resource mobilization. The graphical demonstration may be a good tool for advocacy since stakeholders can easily identify the spatial structure of the disease by watching over the map and may be interested to implement geographically targeted interventions. Furthermore, the study findings may also contribute for the growing body of geostatistical research on TB.

## Methods

### Study area

**Fig. 1:**
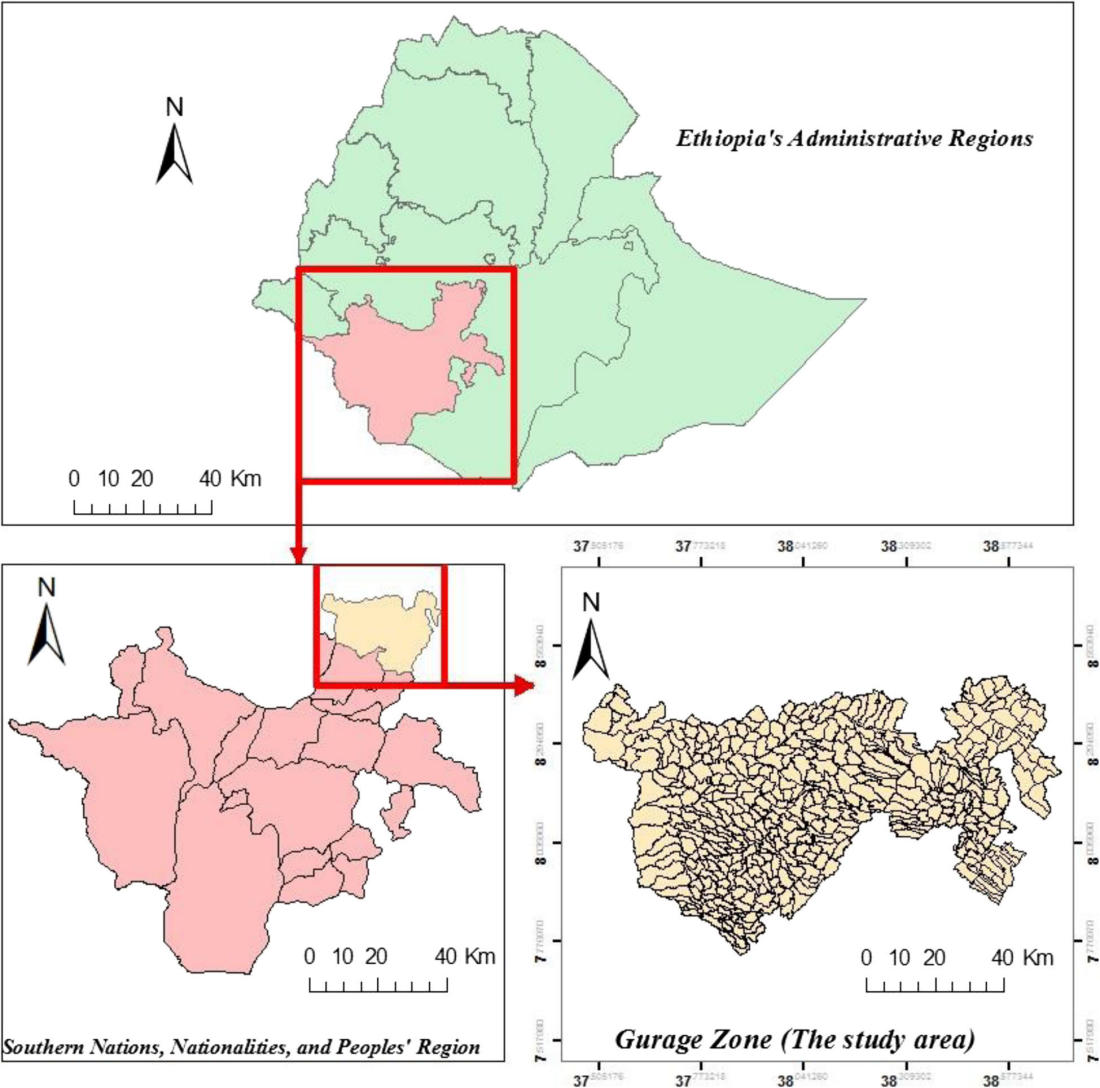
Map of the study area (Gurage Zone). Source: *Tadesse* et al.*, 2018* [17]

This study was conducted in the Gurage Zone in southern Ethiopia, which is located between 7°76′ and 8°45’ N latitude and 37°46′ and 38°71′ E longitude **(**Fig. 1**)**. The zone has 13 districts and two town administrations (at Butajira and Wolkitie). It covers an area of about 5932 km^2^. There are 403 rural and 20 urban kebeles (the smallest administrative units with a population of 5000 on average) in the zone. There were a total of 1,542,131 populations in 2016, about 84% of which live in the rural areas [16].

There are a total of 6 hospitals, 70 health centers, 414 health posts and 92 clinics in the zone that provide TB prevention and control services [16]. The clinics and the health posts provide community education, identify and refer presumptive TB cases to health facilities for further investigation, give *Bacillus Calmette-Guérin* vaccination, contact locating and screening, trace and link lost to follow up cases, and support treatment adherence through female health extension workers. The health centers carry out all activities as health posts and clinics, and additionally provide intensified case finding, sputum microscopy services, provide isoniazid preventive therapy for eligible persons, diagnose and manage adverse drug reactions and other complications, carry out TB/HIV collaborative activities, refer smear negative presumptive TB, extra-pulmonary TB and DR-TB patients to higher level facilities, provide support to health post staff, keep patient records and manage medicines stocks, plan and implement TB infection control. Health centers additionally provide Directly Observed Treatment-Short courses (DOTS) services for patients with DR-TB referred by treatment initiation centers. The hospitals carry out activities as health centers, and additionally provide referral services and admission care for seriously ill TB patients. Selected hospitals provide diagnosis and treatment for DR-TB patients, including inpatient care. The GeneXpert machines are installed at hospital laboratories. Private health facilities are also engaged in TB diagnosis, treatment and/or referral of presumptive TB and DR-TB cases depending on their capacity [17].

### Data sources

The list of health facilities providing DOTS services were obtained from the Health Department database of Gurage Zone. All TB patients who were residents of the zone and registered at the health facilities during January to December, 2016 were included in the study. The patient data were retrieved from the unit TB registers from June to September, 2017. The patients’ addresses were checked for duplication and linked to their true geo-locations. The data on geo-location and population of each kebele in the zone were accessed from the Central Statistical Agency of Ethiopia (CSA). Altitude of each kebele was extracted from ASTER Global Digital Elevation Model V2 [18].

### TB diagnosis and case definition

The diagnostic criteria of the national TB diagnosis guideline of Ethiopia were used to diagnose the TB cases [19].

**Smear-positive pulmonary TB (PTB+):** is diagnosed when at least 2 initial sputum smear examinations are positive for Acid Fast Bacilli (AFB) or 1 smear-positive result for AFB and culture-positive result for *M. tuberculosis* or 1 smear-positive result for AFB and radiographic abnormalities indicative of active TB, in addition to a clinician’s judgment. The regional laboratory carries out external quality assurance on all slides, and provides a feedback to the health facility providing DOTS services.

**Smear-negative pulmonary TB (PTB-):** is diagnosed when there are symptoms evocative of TB, at least 3 smear-negative initial results for AFB, lack of response to antibiotics, smear-negative and radiological abnormalities indicative of pulmonary TB, and judgment of a clinician.

**Extra-pulmonary TB (ETB):** is diagnosed when a specimen from an extra-pulmonary site is culture positive or histo-pathological abnormality from a biopsy, and strong clinical evidence indicative of active ETB. However, because of insufficient laboratories for histo-pathological or culture examinations, most of the health facilities diagnose ETB based on a clinician’s judgment.

**Newly diagnosed case of TB:** is a patient who has never taken anti-TB drugs or taken for less than a month.

**Retreatment TB case:** is a patient who has previous treatment failure, or relapse or default.

### Data quality control

Supervisors and data collectors were trained on the field methods, data extraction and record keeping. The data completeness and consistency was checked page-by-page by health facilities, kebele, and district against unit TB registers.

### Data management and processing

Data were entered, validated, cleaned, and coded using MS Excel (MicroSoft, Redmond, WA, USA). The patients’ data were linked to their true address using CSA codes, and aggregated at kebele level. The aggregated dataset from a total of 423(100%) kebeles were used to examine the actual spatial risk of TB distribution. Then, the aggregated TB datasets co-impacted by geographic factors from sample of 169(40%), 254(60%) and 338(80%) kebeles were used to estimate the spatial risk of TB distribution and associated standard error by using a geostatistical kriging approach (Additional file 1). A simple random sampling technique was used to select the spatial sample kebeles. Geographically weighted central locations were represented by the kebele centroids as coordinates. A table containing the number of TB cases, the population, the coordinates and the prevalence rates (the number of TB cases divided by the population of a given year and multiplied by 100,000) were prepared, and were joined to ArcGIS 10.2.

### Spatial smoothing

Spatial Empirical Bayes Smoothing (SEBS) method was employed in Geographic Data analysis tool (GeoDa) in order to overcome small areas variance instability, which is due to variations in population size as well as few cases of TB in some areas [20]. The population for each kebele was used as a base variable and number of TB cases was used as an event. A queen weights matrix that defines the neighboring kebeles as those with either a shared border or vertex was used for spatial weights [20]. The SEBS method was not applied for the datasets that were used for spatial prediction since the geostatistical kriging would result smoothed estimates by using a weighted linear combination of the known measured values.

### Ordinary kriging

Varieties of kriging have been developed, such as ordinary, universal, simple and indicator. Ordinary kriging was preferred to other types of kriging because it predicts an estimate for unsampled kebele by assuming a constant mean in the local neighborhood of each estimation kebele, which is a characteristic of focal diseases like TB. Besides, it is a good geostatistical method to model data that exhibit spatial trend [21]. It uses a semivariogram model to measure spatial autocorrelation between pairs of prevalence rates as follows [10]:

1 $$ \upgamma \left(\mathrm{h}\right)=\frac{1}{2\mathrm{n}}{\sum}_{\mathrm{i}=1}^{\mathrm{n}}{\left(\mathrm{Z}\left(\mathrm{x}\right)-\mathrm{Z}\left(\mathrm{x}+\mathrm{h}\right)\right)}^2 $$

where *n* is the total number of pairs of sample kebeles, *Z(x)* and *Z(x + h)* are the prevalence rates at any two kebeles *x* and *x + h* separated by distance *h*. Calculations of *γ*(*h*) are repeated for *2 h, 3 h, 4 h, ..., kh*. The models of spatial autocorrelation commonly exhibit similar characteristics, which are called the sill, range, and nugget. The sill is the maximum variability between pairs of prevalence rates. The separation distance at which the sill is reached is termed the range and represents the maximum distance beyond which prevalence rates are spatially independent. The nugget effect refers to the situation in which the difference between prevalence rates taken at sampling kebeles that are close together is not zero. It represents spatial sources of variation at distances smaller than the sampling interval (i.e. spatial variations of prevalence rates at village level, which is a spatial subset of kebele) or measurement error (e.g. passive case detection).

As described in detail previously [12], an unknown prevalence rate ${\widehat{Z}}_u\kern0.5em$at kebele *u* is estimated as a weighted-linear combination of *n* known samples as follows:

2 $$ {\widehat{Z}}_u={\sum}_{\mathrm{i}=1}^{\mathrm{n}}{\mathrm{W}}_{\mathrm{i}}{\mathrm{Z}}_{\mathrm{i}}\kern0.5em $$

where

$$ {\sum}_{\mathrm{i}=1}^{\mathrm{n}}{\mathrm{W}}_{\mathrm{i}}=1 $$

The optimal weights which produce the minimum estimation error in eq. (2) can be determined by using the following simultaneous equations:

3 $$ {\displaystyle \begin{array}{cccccc}{\mathrm{W}}_{\mathrm{i}}\upgamma \left({\mathrm{h}}_{1,1}\right)& +\cdots & +{\mathrm{W}}_{\mathrm{n}}\upgamma \left({\mathrm{h}}_{1,\mathrm{n}}\right)& +& \uplambda =& \upgamma \left({\mathrm{h}}_{1,\mathrm{u}}\right)\\ {}\vdots & \ddots & \vdots & \ddots & \vdots & \vdots \\ {}{\mathrm{W}}_1\upgamma \left({\mathrm{h}}_{\mathrm{n},1}\right)& +\cdots & +{\mathrm{W}}_{\mathrm{n}}\upgamma \left({\mathrm{h}}_{\mathrm{n},\mathrm{n}}\right)& +& \uplambda =& \upgamma \left({\mathrm{h}}_{\mathrm{n}.\mathrm{u}}\right)\\ {}{\mathrm{W}}_1& +\cdots & +{\mathrm{W}}_{\mathrm{n}}& & =& \upgamma \left({\mathrm{h}}_{\mathrm{n}.\mathrm{u}}\right)\end{array}} $$

where *γ*(*h*_*i*,  *j*_) is a semivariogram model which is a function of distance *h*_*i*,  *j*_ between prevalence rates *i* and *j,* and λ is the Lagrange Multiplier to minimize the kriging error. The correlation between prevalence rates *i* and *j* is expected to decrease as their separation distance *h*_*i*,  *j*_ increases. The optimal weights in eq. (2) are calculated as follows:

4 $$ \left[\begin{array}{c}{\mathrm{W}}_1\\ {}\vdots \\ {}{\mathrm{W}}_{\mathrm{n}}\\ {}\uplambda \end{array}\right]={\left[\begin{array}{cccc}\upgamma \left({\mathrm{h}}_{1,1}\right)& \cdots & \upgamma \left({\mathrm{h}}_{1,\mathrm{n}}\right)& 1\\ {}\vdots & \ddots & \vdots & \vdots \\ {}\gamma \left({\mathrm{h}}_{\mathrm{n},1}\right)& \cdots & \upgamma \left({\mathrm{h}}_{\mathrm{n},\mathrm{n}}\right)& 1\\ {}1& \cdots & 1& 0\end{array}\right]}^{-1}\left[\begin{array}{c}\upgamma \left({\mathrm{h}}_{1,\mathrm{u}}\right)\\ {}\vdots \\ {}\upgamma \left({\mathrm{h}}_{\mathrm{n},\mathrm{u}}\right)\\ {}1\end{array}\right] $$

Therefore, ordinary kriging produces an unbiased estimate with minimum variance.

### Ordinary cokriging

Ordinary cokriging is an extension of ordinary kriging method that uses both the spatial autocorrelation for prevalence rate (i.e. the main variable of interest) and the spatial cross-correlations between prevalence rate and geographic variables (i.e. altitude, latitude and longitude) to make estimations of the prevalence rates at unsampled kebeles. The development of the ordinary cokriging system is identical to the development of ordinary kriging system. The mathematical formulation of ordinary cokriging has been described in detail by Yalcin [22].

In this study both ordinary kriging and ordinary cokriging models were tested for the three categories of datasets, and ordinary cokriging models were selected as the best-fitted ones.

### Model selection

In this study the effects of the different types of semivariogram models (i.e., stable, spherical, circular, tetraspherical, pentaspherical, Gaussian, exponential, rational quadratic, K-Bessel, hole effect and J-Bessel), detrending (i.e., neighborhood, global and local), anisotropy (i.e., false and true) and geographic covariates (i.e., longitude, latitude and altitude) on the predictive performance of kriging were checked by using a cross-validation technique. The technique leaves and adds each sample points in the dataset turn by turn to provide pairs of predicted and measured values that can be compared to evaluate the model’s performance. A total of 528 geostatistical kriging models were generated for each category of spatial dataset (i.e., 40, 60 and 80%) (Additional file 2). The final models for each category of the spatial dataset were decided based on the lowest total error, obtained by sorting values of Root-Mean-Square Error (RMSE), absolute value of Mean-Standardized Error (MSE), Root-Mean-Square-Standardized Error (RMSSE) and absolute value of the difference of Average-Standard Error (ASE) from RMSE in ascending order, and then ranking and summing up the ranks. All these errors are expressed by eqs. (5)-(8) below [23]:

5 $$ RMSE=\kern0.5em \sqrt{\frac{1}{n}{\sum}_{i=1}^n{\left[{Z}^{\ast}\left({x}_i\right)-Z\left({x}_i\right)\right]}^2} $$

6 $$ \mathrm{MSE}=\kern0.5em \frac{1}{n}{\sum}_{i=1}^n\left[\frac{Z^{\ast}\left({x}_i\right)-Z\left({x}_i\right)}{\sigma^2\left({x}_i\right)}\right] $$

7 $$ RMSSE\kern0.5em =\kern0.5em \sqrt{\frac{1}{n}{\sum}_{i=1}^n{\left[\frac{Z^{\ast}\left({x}_i\right)-Z\left({x}_i\right)}{\sigma^2\left({x}_i\right)}\right]}^2} $$

8 $$ ASE\kern0.5em =\kern0.5em \sqrt{\frac{1}{n}{\sum}_{i=1}^n{\sigma}^2\left({x}_i\right)} $$

where *σ*^2^(*x*_*i*_) is the kriging variance for location *x*_*i*_, and *Z*^∗^(*x*_*i*_) and *Z*(*x*_*i*_) are the predicted and the sampled values at the location *x*_*i*_, respectively.

### Sensitivity analyses

The Semivariogram Sensitivity tool, which is found under the Geostatistical Analyst toolbox of ArcGIS 10.2, was used to perform sensitivity analyses on the predicted values and associated Standard Errors (SE) by varying the nugget and range within a percentage of the original values. The outputs of the analyses were a table indicating which parameter values were used and what the resulting predicted and standard error values were. Small fluctuations in the output with small changes in the input parameter values indicate more confident predictions which can be used to make decisions.

### Risk measurement

The prevalence rate was used as a proxy variable to estimate the risk of TB in the study area. The estimated risk surface was categorized as *a low risk area* where the prevalence rate was below and equal to 100 cases per 100,000 population, and *a high risk area* where the prevalence rate was above 100 cases per 100,000 population [20].

## Results

### Patient characteristics

A total of 1626 TB cases were diagnosed during January to December, 2016. Only 1.6% of them were excluded from the final analyses because of incomplete addresses or being outside of the study area. Out of 1601 cases included in this study 57.5% were males and 42.5% were women, yielding a male to female ratio of 1.3:1. The mean age with a standard deviation was 36 ± 17 years for all cases, 34 ± 16 years for males and 38 ± 17 years for females. The majority, 89.6%, of the cases were newly diagnosed, while 10.4% were retreatment cases. Of the cases 41.2% were PTB+, 31.9% PTB- and 26.9% ETB. Residentially, 86.6% were from rural areas.

### The actual spatial risk of TB distribution

**Fig. 2:**
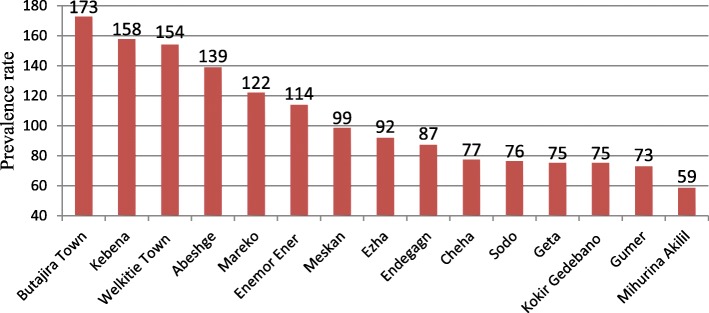
TB prevalence rates by districts in Gurage Zone

**Fig. 3:**
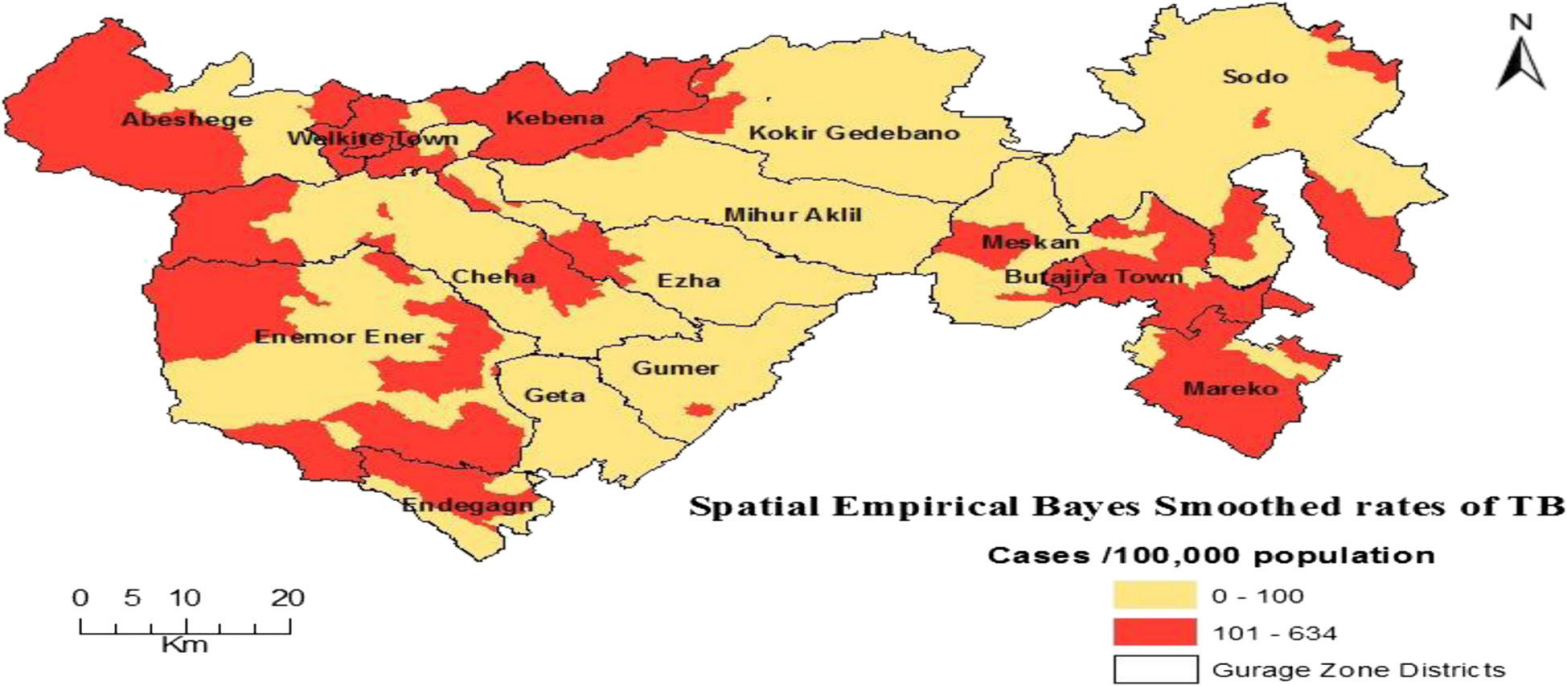
The SEBS rates of TB in Gurage Zone. Source: *Authors own data*

The risk distribution of TB varied from 59 to 173 cases per 100,000 population across districts of the Gurage Zone (Fig. 2). Moreover, the smoothed rates of TB varied from zero to 634 cases per 100,000 population across kebeles of the zone. High risk of TB was observed at northwest, western, southwest and southeast parts. The risk distribution crossed between high and low at west-central parts (Fig. 3).

### The best-fitted models

The best geostatistical kriging models were decided to be: 1) Pentaspherical semivariogram, local detrending, true anisotropy and altitude and latitude covariates for modeling with 40% of spatial dataset, 2) Rational Quadratic semivariogram, local detrending, true anisotropy and altitude and latitude covariates for modeling with 60% of spatial dataset, and 3) K-Bessel semivariogram, global detrending, true anisotropy and altitude and latitude covariates for modeling with 80% of spatial dataset. The detrending pattern of the models changed from local to global as the size of spatial dataset increased. Moreover, the models predictive accuracies also improved as the size of spatial dataset increased, which was indicated by 0 MSE, 1 RMSSE, and ASE approached RMSE (i.e., the variability in prediction is correctly assessed) (Table 1).

**Table 1:** Comparison of cross-validation statistics for TB spatial datasets in Gurage Zone, Southern Ethiopia, 2017

Cross-validation statistics	Ordinary cokriging models		
	With 40% dataset	With 60% dataset	With 80% dataset
MSE	0	0	0
RMSSE	1	1	1
RMSE	89	88	87
ASE	93	87	87

### Sensitivity analyses outputs

The parameter values for nugget and range from the input geostatistical model sources were 8123.85 and 72,891.45 for the model with 40% spatial dataset, 7178.77 and 78,808.04 for the model with 60% spatial dataset, and 7210.46 and 78,767.27 for the model with 80% spatial dataset, respectively. Five random nugget and range values that were found within 10% of the input models’ nugget and range values were calculated for each dataset and used as input parameters. There were only small fluctuations in the prediction outputs for the corresponding input parameters, indicating more accurate predictive performance of the models (Table 2).

**Table 2:** The semivariogram sensitivity analyses results for TB spatial datasets in Gurage Zone, Southern Ethiopia, 2017

Model	Random Parameter	Prediction	SE	Nugget	Range
Modeling with 40% dataset	Nugget	79.00	22.95	7898.75	72,891.45
	Nugget	78.99	23.34	8170.68	72,891.45
	Nugget	79.01	22.66	7702.39	72,891.45
	Nugget	79.01	22.35	7467.51	72,891.45
	Nugget	79.02	22.47	7572.18	72,891.45
	Range	78.99	23.18	8062.43	76,769.04
	Range	78.65	23.19	8068.52	79,894.51
	Range	78.66	23.17	8050.92	72,346.21
	Range	78.66	23.17	8051.90	72,671.44
	Range	78.65	23.19	8068.39	79,821.99
Modeling with 60% dataset	Nugget	67.35	21.45	6995.20	78,808.04
	Nugget	67.63	20.94	7360.37	78,808.04
	Nugget	67.57	21.05	7284.92	78,808.04
	Nugget	67.66	20.90	7425.62	78,808.04
	Nugget	67.68	21.24	7669.06	78,808.04
	Range	67.44	21.16	7018.50	80,198.91
	Range	83.57	21.03	6920.55	73,192.36
	Range	67.55	21.00	7076.75	85,175.07
	Range	77.54	20.82	6979.91	77,256.03
	Range	67.55	21.00	7076.00	85,106.29
Modeling with 80% dataset	Nugget	77.35	20.49	7017.76	78,767.27
	Nugget	77.46	20.86	7356.95	78,767.27
	Nugget	77.51	21.51	7865.18	78,767.27
	Nugget	77.20	20.02	6603.89	78,767.27
	Nugget	77.45	20.84	7332.31	78,767.27
	Range	77.82	20.60	7212.61	80,939.15
	Range	77.82	20.60	7212.40	80,888.19
	Range	74.71	19.43	7172.59	72,233.82
	Range	74.71	19.44	7179.62	73,658.34
	Range	77.82	20.61	7221.91	83,206.47

### The estimated spatial risk of TB distribution

**Fig. 4:**
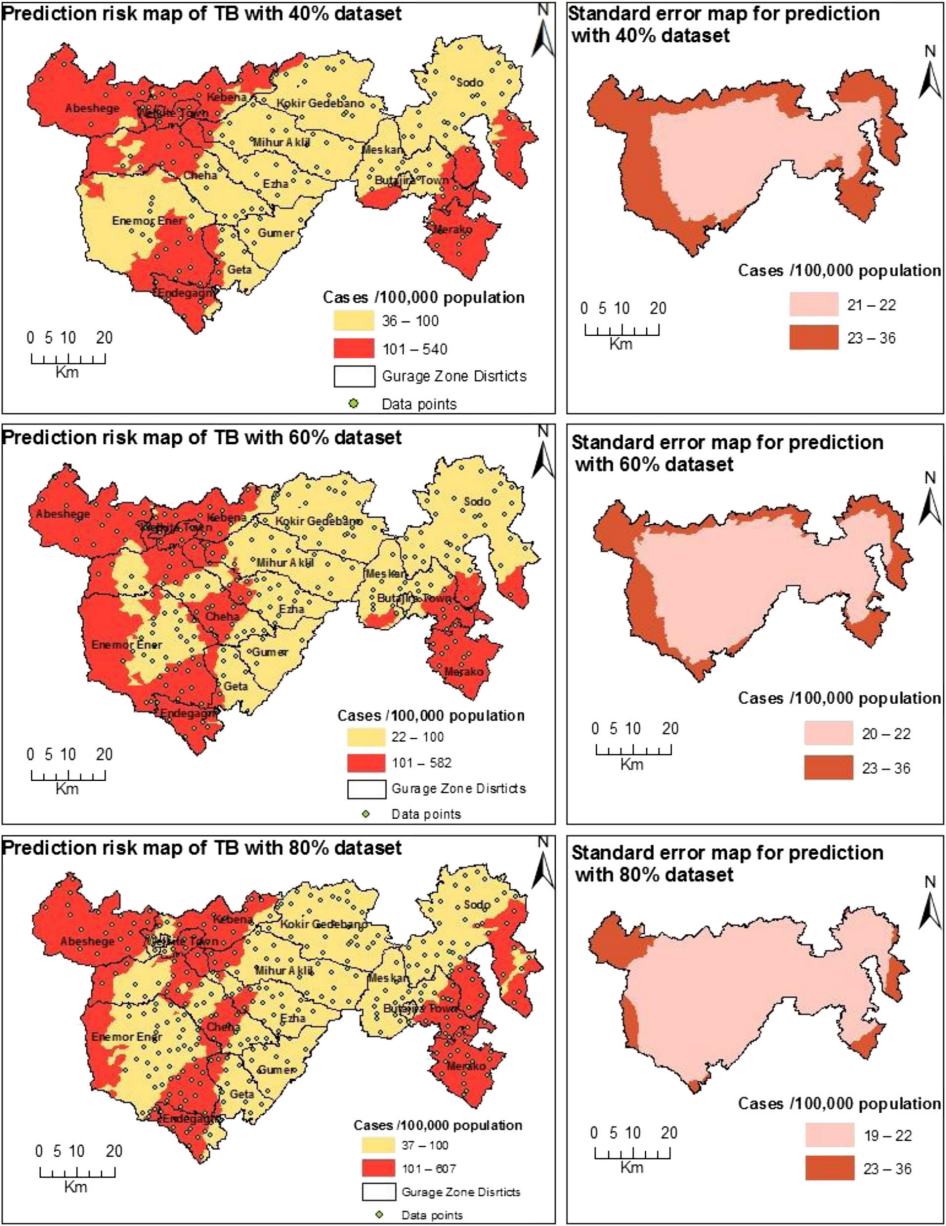
The prediction risk maps of TB and associated standard error maps in Gurage Zone. Source: *Authors own data*

The ordinary cokriging models with 40, 60 and 80% of the spatial datasets estimated high risk of TB at northwest, western, southwest and southeast parts of the Gurage Zone. The risk distribution crossed between high and low at west-central parts**.** Moreover, the models estimated high uncertainties of prediction at border areas of the zone, the magnitude of which decreased as the spatial dataset increased (Fig. 4).

### Comparison between estimated and actual spatial risk of TB distribution

The three estimation models identified areas for high risk of TB at locations that were closely similar to the actual high-risk areas, with reasonable predictive accuracies. These locations included northwest, western, southwest and southeast parts of the Gurage Zone. The risk distribution crossed between high and low at west-central parts (see Figs 3 and 4 above**)**.

## Discussion

This study has underscored that the geostatistical kriging approach can be applied to estimate the spatial risk of TB distribution in settings where spatially limited data are available. The estimation models indicated that there was spatial heterogeneity in the risk of TB distribution in the Gurage Zone, indicating the disease did not affect all of the communities in the area with the same severity. The risk was higher in northwest, western, southwest and southeast parts of the zone. However, the risk distribution interlocked between high and low at west-central parts. Evidences have revealed that differences in underlying socioeconomic, climatic and geographic conditions, and uneven allocation of public health resources could contribute for the spatial heterogeneity in the risk of TB distribution [3, 24, 25]. Moreover, the cross-border population movements from the neighboring border areas could also facilitate the high transmission of TB, especially at border areas of the zone [26–30]. Therefore, the estimated risk map of TB may help local public health authorities prioritize locations that required immediate interventions.

This study revealed that cokriging with altitude and latitude were the best geostatistical models, which suggested that including these covariables improved the predictive accuracies of the models. This reflects that geographical factors can affect the risk distribution of TB in the Gurage Zone. Previous studies have highlighted that the geographical factors had explicit impacts on the risk distribution of TB [3, 24, 25, 31, 32]. Thus, impacts of geographical factors on TB prevention and control should be evaluated, and interventions should be formulated based on geographical features.

In the present study the prediction standard error values were relatively higher in the western, northwest, southwest and southeast parts than in the others. This could be due to the fact that the spatial distribution of TB risk was higher in the western, northwest, southwest and southeast parts of the study area and kriging method underestimated the higher values [3]. The other reason could be that the majority of the spatial sample points were sparser in areas with higher prediction standard error [3]. The lack of data point beyond the borders of the study area could also explain the higher prediction standard error [12]. Therefore, it would be better to take sample data points with better spatial distribution beyond or on the boundaries of these locations in order to obtain more accurate and stable kriging surface estimates.

This study has also practical implications to TB prevention and control programs in low-income countries, where obtaining spatially complete TB data is difficult. The recent advancements in geostatistical modeling techniques and increasing availability of public health data from the national prevalence surveys, demographic and health system surveys and health facilities will be the good opportunities for epidemiologists working in such settings to predict the spatial risk of TB distribution and associated prediction uncertainty at non-surveyed locations [3, 8, 11]. The resulting prediction risk map may allow them measure burden of the disease at all locations, identify high-risk geographical areas for targeted interventions, and evaluate the impacts of intervention programs. This will be useful for optimal utilization of the scarce public health resource.

This study has some limitations. The estimated risk of TB might be underestimated in some areas because the study did not include those patients who would remain undiagnosed for the disease, and those diagnosed and treated at health facilities outside the study area. The modifiable areal unit problem might arise due to the spatial unit of data aggregation. However, the spatial unit of analysis used in this study was the finest resolution available, kebele, which was also the spatial unit used for healthcare planning in the study area. The denominator population numbers could be affected by uneven population growth across the study area since the numbers were projected from the 2007 census [33].

## Conclusion

This study has underlined that the geostatistical kriging approach can be applied to estimate the spatial risk of tuberculosis distribution in data limited settings. The estimation results may help local public health authorities measure burden of the disease at all locations, identify geographical areas that require more attention, and evaluate the impacts of intervention programs.

## Additional files

Additional file 1: The aggregated datasets for the study. (XLSX 68 kb)

Additional file 2: The geostatistical kriging models. (XLSX 232 kb)

## Abbreviations

AFB: Acid Fast Bacilli
ASE: Average Standard Errors
CSA: Central Statistical Agency of Ethiopia
DOTS: Directly Observed Treatment-Short courses
DR-TB: Drug Resistant TB
ETB: Extra-pulmonary TB
MSE: Mean Standardized Error
PTB-: Smear-negative pulmonary TB
RMSE: Root-Mean-Square Error
RMSSE: Root-Mean-Square Standardized Error
TB: Tuberculosis
TB/HIV: Human Immunodeficiency Virus (HIV)-associated TB
PTB +: Smear-positive pulmonary TB.
SEBS: Spatial Empirical Bayes Smoothing
SE: Standard Errors
